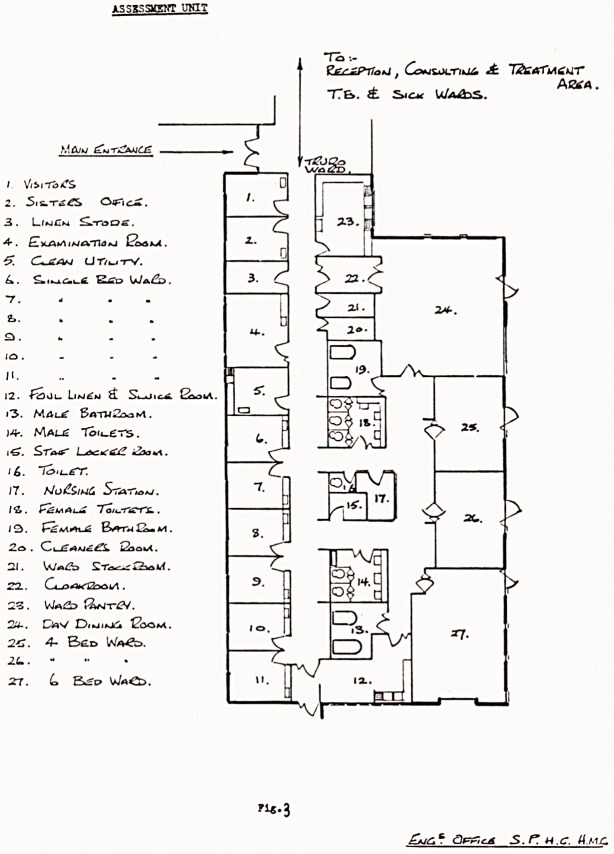# Assessment Unit for the Mentally Retarded

**Published:** 1971-04

**Authors:** J. Jancar

**Affiliations:** Consultant Psychiatrist, Stoke Park Hospital Group, Bristol


### Assessment Unit for the Mentally Retarded

**by J. Jancar, M.B., B.Ch., B.A.O., D.P.M.**

Consultant Psychiatrist, Stoke Park Hospital Group, Bristol

**INTRODUCTION**

The assessment of mentally retarded patients has recently become an important issue among the people working in the field of mental retardation. Various proposals as to the staffing and siting of assessment units have been suggested and discussed.

We at Stoke Park have been concerned for some time with the question of assessment and we have come to the conclusion that the problem of mentally retarded patients can best be resolved by:

1. full mental and physical assessment
2. proper placement and 3. regular re-assessment.

Full assessment can only be made over a period in a suitable unit staffed with a multidisciplinary team. When the assessment is completed. the patient can be properly placed back in his family or in day or residential care in the community or in hospital.

Sometimes all of these placements can be utilized in the same case to provide proper care, training, treatment and rehabilitation of an individual patient, e.g. the patient lives with his family, attends the community day centre and, over the weekend and when the training centre is closed, becomes a day patient at the hospital.

Wherever the patient is placed, regular re-assessment is essential, so that progress or relapse can be followed and to ensure that the patient is still properly placed and that all the necessary services are provided for him.

We are presenting here two reports on assessment units for the mentally retarded. Firstly, a six years' survey of a unit — a pilot scheme which we started at Hanham Hall in October, 1961 — and secondly a six months' report on the new unit at Stoke Park Hospital, which opened in September, 1970, as a result of the Hanham Hall experiment.

### HANHAM HALL EXPERIMENT *

The Bristol Assessment Clinic for the mentally retarded was opened ten years ago on the premises of the Local Health Authority. We found that proper assessment of some patients was not possible since the time required for assessment and necessary further investigations was not available. Fortunately we were able to use a newly opened seven-bedded extension of the sick ward at Hanham Hall (one of the hospitals for male patients in the Stoke Park Hospital Group) as an assessment unit, from October, 1961, onwards. During the subsequent six years, 71 patients have been admitted there for assessment, each staying on average for four weeks.

Most of these patients were referred by the Bristol and Gloucester Assessment Clinics (the latter having been opened six years ago), although some patients were referred directly from Local Health Authorities or their hostels, domiciliary visits, courts, consultants from other specialities, general practitioners and educational authorities. Of the 71 patients assesed, 34 were admited from Bristol, 22 from Gloucester, 10 from Wiltshire, 2 from Bath, 2 from Somerset and 1 from Gloucester City Local Health Authorities.

**REASONS FOR REFERRAL TO THE ASSESSMENT UNIT**

The majority of the patients were referred to the unit because of behaviour disorders, either at home or at training centres. Some were referred because of mental or physical deterioration or both, whilst others were admitted because of superimposed psychotic episodes. A few came to the unit because of illness or death of parents or relatives, for assessment and future placement, as were patients referred from the courts. Epileptic patients were also admitted for assessment, but in addition, their anticonvulsant treatment was adjusted to control their fits, or to change their drugs when side effects became apparent. In many cases admission for assessment had been requested during holiday periods to give relatives a well deserved break.

As Hanham Hall Hospital provides 230 beds for severely subnormal adult males only, admissions had to be selected accordingly. The three youngest patients were 17 years of age, the mean chronological age of all admissions being 30.6 years. The I.Qs. ranged from 15 - 114, with a mean value of 41.4.

On admission every patient underwent a very detailed medical and mental routine examination (Fig. 1). Further investigations of relevant findings were carried out by the hospital medical staff, or where necessary, by specialists from other hospitals. Nursing, occupational therapy, medical ancillary and other staff also assessed the patients and reported their observations. The well established hospital industrial and occupational therapy department was very useful in the assessment of the skill of individual patients (Cameron and Nicoll, 1961).

*Presented at the 7th International Congress on Mental Health, London, August, 1968.

STOKE PARK GROUP HOSPITAL MANAGEMENT COMMITTEE

ASSESSMENT UNIT

Patient's name ____________ Date of Birth ____________

Date of Admission ____________ Date of Discharge ____________

Reg. No. ____________

| | Date of investigation | Date report received |
|---|---|---|
| ROUTINE INVESTIGATIONS | | |
| 1. Physical examination | | |
| 2. Neurological examination | | |
| 3. Ophthalmological examination | | |
| 4. Dental examination | | |
| 5. Psychological assessment | | |
| 6. Educational assessment | | |
| 7. Occupational and industrial therapy assessment | | |
| 8. Physiotherapist's assessment | | |
| 9. Speech therapist's assessment | | |
| 10. Chiropodist's assessment | | |
| 11. X-ray - chest (and skull in case of epileptics) | | |
| 12. Full face and profile photographs | | |
| 13. Urine - routine ward examination | | |
| 14. Paper chromatography | | |
| 15. Dermatoglyphs | | |
| 16. Full blood count | | |
| 17. W.R. and Kahn | | |
| SPECIAL INVESTIGATIONS (Where ringed by Consultant) | | |
| 18. E.N.T. examination | | |
| 19. Audiometry | | |
| 20. E.E.G. | | |
| 21. Ketosteroid estimation | | |
| 22. Serum cholesterol estimation | | |
| 23. Glucose tolerance test | | |
| 24. Chromosomal investigation | | |
| 25. Skeletal survey | | |
| 26. Clinical photography | | |
| ADDITIONAL INVESTIGATIONS | | |

Date .................... Consultant Psychiatrist ....................

ASSESSMENT UNIT INVESTIGATIONS FORM

**Fig. 1.**

The multi-disciplinary clinical examinations and investigations revealed the following abnormalities:

(a) **E.E.G.:** There were 14 known epileptic patients admitted, abnormal recordings were found in a further 14 patients.

(b) **X-Rays:** Skeletal abnormalities were detected in 13 patients, whilst the choroid plexus were calcified in two patients.

(c) **Abnormal sexual characteristics:** Female distribution of pubic hair was recorded in six male patients, of which one also had gynecomastia and another had a very low urinary excretion rate of 17-ketosteroids (17-oxosteroids).

(d) **Glucose tolerance:** Glucose tolerance curves revealed abnormally increased tolerance in five patients (i.e. flattened curves).

(e) **Obesity:** Marked obesity, at present of unknown origin, was noted in two brothers.

(f) **Urine chromatography:** Urine chromatography was carried out on all patients in which the cause of mental retardation was not known. Abnormal aminoacid excretion patterns were found in two patients.

(g) **Blood pressure:** The blood pressure was found to be within the normal limits for the patients' ages in all the patients except for two suffering from hypertension.

(h) **Deafness:** Hearing was also investigated and two cases of very severe deafness were discovered.

(i) **Other anomalies:** Single cases of the following anomalies were detected during the course of investigations:

Abnormal E.C.G., spasticity, alopecia totalis, Scandinavian type of scabies, ichthyosis, ankylosing spondylitis, anaemia, cataracts, ptosis of eyelids, cleft palate, absent uvula, Spigelian-ventral hernia, hiatus hernia, cystitis with haematuria, threadworms and abnormal dermatoglyphic pattern (not including abnormal dermatoglyphs found in the patients suffering from Down's syndrome).

(j) **Dental care:** All the patients were examined by the dental surgeon to the Group. He carried out all necessary treatment to teeth and gums and, in addition, made dental impressions from patients with rare or as yet unknown disorders for future studies and analyses.

### SYNDROMES AND OTHER DEFINITE CAUSES OF MENTAL RETARDATION

1. Down's syndrome. 13 cases — 11 were diagnosed clinically, one was confirmed by chromosomal analysis and chromosomal analysis revealed a further case as a mosaic Down's syndrome.
2. Ring chromosome 18 — one case.
3. "Cretinism" — two cases.
4. Marfan's syndrome — two cases.
5. Post-meningitic encephalopathy — two cases.
6. Post-vaccinial encephalopathy — two cases.
7. Post-influenzal encephalopathy — one case.
8. Deaf and dumb — one case.
9. Toxoplasmosis—one case, confirmed by postitive antibody titres in the serum and by the presence of calcification in the brain. There were also two cases of microcephaly, but we were unable to establish the aetiology of these. A few cases of "brain injured patients" are not included under this heading as the diagnoses were either only tentative, or the clinical histories were too vague or incomplete. The percentage of syndromes known to cause mental retardation occurring in the patients studied is more or less in keeping with present knowledge (Eastham and Jancar, 1968).

### FAMILY HISTORY

When patients have been admitted to the unit all the available information of the physical and mental health of both the patients and their relatives has been collated. The records from the family doctors, local health authorities, hospitals, educational and other authorities have been studied and, wherever possible, the relatives have been interviewed. This had made possible not only a more accurate assessment of the patient and the diagnosis of the patient's illness, but has also encouraged us in the study of familial disorders which should eventually lead to further advances in the discovery of causes of mental retardation and their treatment and prevention.

Out of 71 mentally retarded patients, it was found that four have relatives with mental illness and three have more than one mentally retarded sib, whilst one has a deaf and dumb sister. One patient had thirteen sibs, several of whom were deaf or partially deaf. The parents of a further patient were found to be second cousins.

### ON DISCHARGE

When a patient was discharged, a detailed report was prepared, which included the results of the multidisciplinary physical and mental investigations, treatment and recommendations of future treatment, rehabilitation and placing. This report was sent to the patient's own doctor, with copies to the appropriate local health authority and other referring authorities or doctors.

### FOLLOW-UP AFTER DISCHARGE

1. **Hospitals :** Of the patients described in this series, 21 required permanent admission to a hospital for the mentally subnormal, after they had been assessed in the unit, in most cases as a result of further mental or physical deterioration. Three of these patients died in hospital, one from post-operative hypotension, one from coronary artery thrombosis and a third from acute pulmonary oedema associated with cardiac failure.

2. **Hostels :** Three of the patients are resident in local health authority hostels for the mentally subnormal.

3. **Day Hospitals :** Two patients are attending hospital on a daily basis.

4. **Special Hospitals :** One patient, soon after discharge from the assessment unit, assaulted two women and was, therefore, admitted to a special hospital.

5. **Home, Training Centres and Assessment Clinics :** The remainder of the patients are at home and attend the training centres of the local health authorities, being seen periodically at the assessment clinic. Twelve of these patients were admitted to Hanham Hall for short term care during training centre holiday periods, or as a result of illness or holidays of relatives.

### DISCUSSION

The success of the assessment unit soon became apparent. As a result patients were admitted for further assessment whenever a bed became available, through the Stoke Park Hospital Group. Up to October, 1967, a total of 121 patients, including both children and adults, males and females of many different ages and I.Q.s, were referred.

The benefits derived from the assessment unit are as follows :

1. **The patient :** The patient is fully examined, assessed and treated. His future rehabilitation is planned more advantageously and the available facilities are used for his benefit. Admission enables the patient to lose his fear and prejudice against hospitals and their staff, which is most valuable in the event of emergency readmission or permanent hospital care. He also learns to live in the new environment and appreciates his home environment and its advantages. As it should be, the unit is of greatest benefit to the patient.

2. **Parents and relatives :** Parents and relatives learn the truth about their children and how to adjust their lives to physical and mental limitations. They appreciate the link with hospital staff, which enables them to seek advice and guidance in the care of their retarded children, in regard to diet, hygiene and other daily problems. Elderly parents obtain comfort from the knowledge that in the event of illness or death, their children will be cared for in the hospital or elsewhere. There are no fixed visiting hours laid down in the assessment unit and, if health permits, patients are allowed to go out with their relatives. Thus the family unit is maintained and the hospital for the mentally retarded is beginning to be regarded as just another hospital.

3. **The community :** The assessment unit results in better liaison with all the community services available for the mentally retarded. Since the reports sent out by the unit contain all the available data at the time of discharge, repetition of investigations and examinations is avoided and the planning of future rehabilitation, treatment and placing becomes more uniform, with more effective use of available manpower.

4. **The nursing staff and other ancillary staff :** The hospital staff is given a stimulus, challenge and opportunity to learn about advances in the study of mental retardation and to observe and carefully record the anomalies demonstrated in patients by the medical staff. The Assessment Unit builds up a team spirit and this is essential for the success of any such unit. Inevitably they are brought into closer contact with parents, the officers of the community services and their colleagues in other hospitals.

5. **Long-stay patients :** Patients who are in permanent care in the hospital, because of the activities of the assessment unit, see new people and new activities. They often take an interest in new admissions, which is of great help both in reassuring the new patients and in giving the long-stay patients a sense of purpose and responsibility. Following admission of patients to the unit, their relatives have often joined the League of Friends of the Hospital, which in turn helps the long-stay patients.

6. **Medicine :** Finally, the assessment unit has brought together all branches of medicine in the search for causes, treatment and prevention of mental retardation. Careful examination of patients and recording of normal and abnormal data, has resulted in the collection of valuable material for future research. Good example of this cooperation are two recent studies on plasma viscosity and serum cholesterol in mentally retarded patients, when the special investigations were included with routine blood examinations (Eastham and Jancar, 1965 and 1968).

As a result of referral elsewhere in the Stoke Park Hospital Group, the first case of Rubinstein-Taybi's syndrome in this country (Jancar, 1965, a) and a rare case of Cerebro-metacarpo-metatarsal dystrophy (Jancar, 1965, b) were reported. Subsequently five more cases of the latter syndrome were discovered.

### PERSONAL FILE

During the collection of relevant data and completion of medical histories of patients admitted to the Assessment Unit, it became apparent that on the one hand many investigations and tests were reduplicated unnecessarily, whilst on the other hand, many useful reports of investigations were either lost or untraceable. Delay and unnecessary effort resulted from the considerable correspondence dealing with the past histories of some patients. Many hospitals and institutions destroy the files of patients after a certain time has elapsed and it is therefore suggested that a nationally or, better still, internationally agreed personal file should be designed to be provided for every mentally retarded patient. The file should contain only relevant reports of mental tests, medical and other data, and should be kept by the person in charge of treatment of the patient at that time. Since computers are being used increasingly in research into mental retardation, the file should be designed with this in view.

### CONCLUSION

The pilot assessment unit at Hanham Hall and the subsequent expansion of the assessment scheme throughout the Stoke Park Hospital Group, including children and adults, males and females, enabled us to gain further valuable experience in the study and care of mentally retarded patients. As a result of this successful project, a permanent 20-bedded assessment unit for children and adults of both sexes was built at Stoke Park Hospital. In the meantime the above arrangements continued.

### NEW ASSESSMENT UNIT AT STOKE PARK HOSPITAL

### (the first six months)

This unit is part of a larger building programme for the replacement of old and unsuitable buildings. (Figs. 2 and 3). The unit opened in September, 1970. It is a single storey building, linked with out-patients, sick ward and departments for staff in professions supplementary to medicine. There are six single rooms, two four-bedded and one six-bedded room.

Fig. 3

Fig. 2

### STAFFING OF THE UNIT

(a) **Medical:** The beds in the unit are divided between the three consultant psychiatrists and medically covered, by day, by one part-time senior registrar, one registrar and, at night and weekends, by one of the assistant psychiatrists or general practitioners.

(b) **Nursing Team:** This consists of one sister, one staff nurse, two student nurses and two nursing assistants by day and at night by one state enrolled nurse, one nursing assistant, with night nursing officer coverage. There are also a full-time occupational therapist and a school teacher in daily attendance. All the staff in the supplementary medical professions appointed to the group are also actively involved in the unit. The services not available in the hospital are, as before, provided by other hospitals in Bristol, especially by Frenchay Hospital and the Burden Neurological Institute.

Visiting consultants for other branches of medicine provide the necessary expert help in the investigations and treatment of the patients in the assessment unit. The unit is also visited weekly by the dietician who supervises the patients' special diets.

### REFERRAL OF THE PATIENTS

The majority of patients were referred through the Bristol and Gloucester Assessment Clinics and through the newly opened assessment clinic on the premises of the assessment unit, serving the area of South Gloucestershire, Bath and part of Wiltshire. Patients were directly referred from the Bristol Royal Hospital for

Sick Children, the Children's Department of Local Health Authorities, general practictioners and one from Sandhill Park and another from Hortham-Brentry Hospital Group.

### SEX, AGE AND AVERAGE STAY

Since the opening six months ago, forty-six patients — twenty-one males and twenty-five females — have been admitted for assessment. Three came as day patients. The ages varied from six months to seventy-three years four months. The mean chronological age of all admissions was eighteen years and one month. On the average patients stay in the unit for about four weeks. A few patients have stayed longer because of further tests and observations.

### MENTAL STATE

Most of the patients admitted were mentally severely retarded. I.Q's ranged from below 20 to 72.

Various neurotic traits superimposed on mental retardation were observed and one case of senile dementia was noted. One patient, suffering from vascular degeneration developed psychotic episodes.

### PHYSICAL ANOMALIES

There were fourteen known epileptics and five patients had abnormal E.E.G. recordings. The treatment of the epileptics was reassessed and in a number of them the gums required dental treatment.

Routine blood examination revealed Hb. below 80% in nine patients. Two patients suffered from myoclonic movements, the cause of which is being further investigated. The E.C.G. in two patients was abnormal. Conductive deafness associated with chronic naso-pharyngeal infection was detected in one patient and central aphasia was observed in another. The blood pressure of a female patient was stabilized during her stay in the unit and extensive dental treatment was given under general anaesthetic in another case. On physical and X-ray examination a number of skeletal abnormalities were noted. Dermatoglyphs were taken from all the patients admitted for assessment.

### SYNDROMES OBSERVED IN THE UNIT

Four patients with Down's syndrome each presented a particular problem. The first suffered from Fallot's tetralogy, the second from persistent vomiting, the third from severe behaviour disorder and the fourth from possible epileptic attacks and herniation through the sinus of Morgagni.

A case of congenital Leber's amaurosis was admitted to the unit but died seven days after admission from broncho-pneumonia.

Smith-Lemli-Opitz syndrome was diagnosed in a female baby (Dallaire, 1969).

A patient suffering from ? Rubella syndrome (Cooper and Krugman, 1966) and another from ? "Male Turner" syndrome (Heller, 1965) are still being investigated.

### DISCHARGE AFTER THE ASSESSMENT

Of the forty-six patients, six are still undergoing assessment and one died, as mentioned above. Twenty-five returned to the family or community care. Twelve patients were admitted to Stoke Park Hospital for permanent care. Ten of these are very severely mentally and physically handicapped babies and children under five years of age, who were referred from the Children's Hospital or whose parents are unable to look after them. One female patient who was transferred to our tuberculosis ward, suffered in the past from pulmonary tuberculosis and has recently developed nodular vasculitis possibly of tuberculous origin, and a case of senile dementia was transferred to the geriatric ward at Stoke Park.

Two patients referred from hospitals for the mentally retarded returned to their respective hospitals. The patients discharged home or in the community will be followed up at Assessment Clinics. There are twenty-five patients on the waiting list for the assessment unit. When the waiting list is cleared we are hoping to use a few beds for temporary care to give relief to the parents of their mentally retarded children during holidays or through illness.

In future all new admissions to the Stoke Park Hospital Group will be admitted first to the Assessment Unit for full assessment before being placed in a particular ward. Reassessment, when required, of the patients from the hospitals in the group and from the community will also take place in the assessment unit when a vacancy becomes available.

### CONCLUSION

The new assessment unit at Stoke Park, in its six months' existence, confirmed the value of the Hanham Hall experiment and the future need for such a unit as long as the problem of mental retardation exists, which in spite of great advances in prevention and treatment, is likely to be for many years to come.

### ACKNOWLEDGEMENTS

I wish to thank the South Western Regional Hospital Board and Stoke Park Hospital Group Management Committee and their officers for their support of the Hanham Hall Experiment and for the planning and building of the new Assessment Unit at Stoke Park Hospital, Dr. W. A. Heaton-Ward, Consultant Psychiatrist-in-Charge, for permission to include his patients in the survey and for most helpful discussions, Professor C. E. Dent, F.R.S., University College Hospital Medical School, London and Professor L. S. Penrose, F.R.S., the Kennedy-Galton Centre, Harperbury Hospital, Hertfordshire, for their expert advice and help with special investigations, and Dr. Ruth Walters, Consultant Psychiatrist, for allowing me to include in the study her patients from the new Assessment Unit.

I also thank the Medical Officers of Health and their staff from Bristol, Gloucestershire, Wiltshire, Bath, Cheltenham, Gloucester City and Somerset for constant help and cooperation when searching for relevant information, Stoke Park medical colleagues, nursing, occupational therapy, teaching and other hospital staff, who always willingly accepted extra burdens on their already full programme of routine work, for their careful observations and collections of specimens, medical colleagues from many departments of Bristol Hospitals, especially Frenchay Hospital, for undertaking extra work with sometimes very difficult, time consuming examinations and tests, and Mrs. K. J. Hiscock for the secretarial work.

### REFERENCES

1. **Cameron, F. and Nicoll, S.** (1961) Industrial and Social Therapy; An Experiment. Nursing Times.
2. **Cooper, L. Z. and Krugman, S.** (1966) Diagnosis and Management: congenital rubella. Pediatrics, **37,** 335.
3. **Dallaire, L.** (1969) Syndrome of Retardation with Urogenital and Skeletal Anomalies (Smith-Lemli-Opitz Syndrome); Clinical features and mode of inheritance. Journal of Medical Genetics, **6,** 113.
4. **Eastham, R. D. and Jancar, J.** (1965) Plasma Viscosity in cases of Severe Mental Subnormality. American Journal of Mental Deficiency, **69,** 502.
5. **Eastham, R. D. and Jancar, J.** (1968) Serum Cholesterol in Mental Retardation. Proc. 1st Cong. of Int. Ass. for the Scientific Study of Mental Deficiency, Montpellier, 1967.
6. **Eastham, R. D. and Jancar J.** (1968) Clinical Pathology in Mental Retardation. John Wright & Sons Ltd., Bristol.
7. **Heller, R. H.** (1965) The Turner phenotype in the male. Journal of Pediatrics, **66**, 48.
8. **Jancar, J.** (1965a) "Rubinstein-Taybi's Syndrome". Journal of Mental Deficiency Research, **9,** 266.
9. **Jancar, J.** (1965b) Cerebro-Metacarpo-Metatarsal Dystrophy (Pseudo - Pseudo - Hypoparathyroidism) with Chromosomal Anomaly. Journal of Medical Genetics, **2,** 32.

## Figures and Tables

**Fig. 1. f1:**
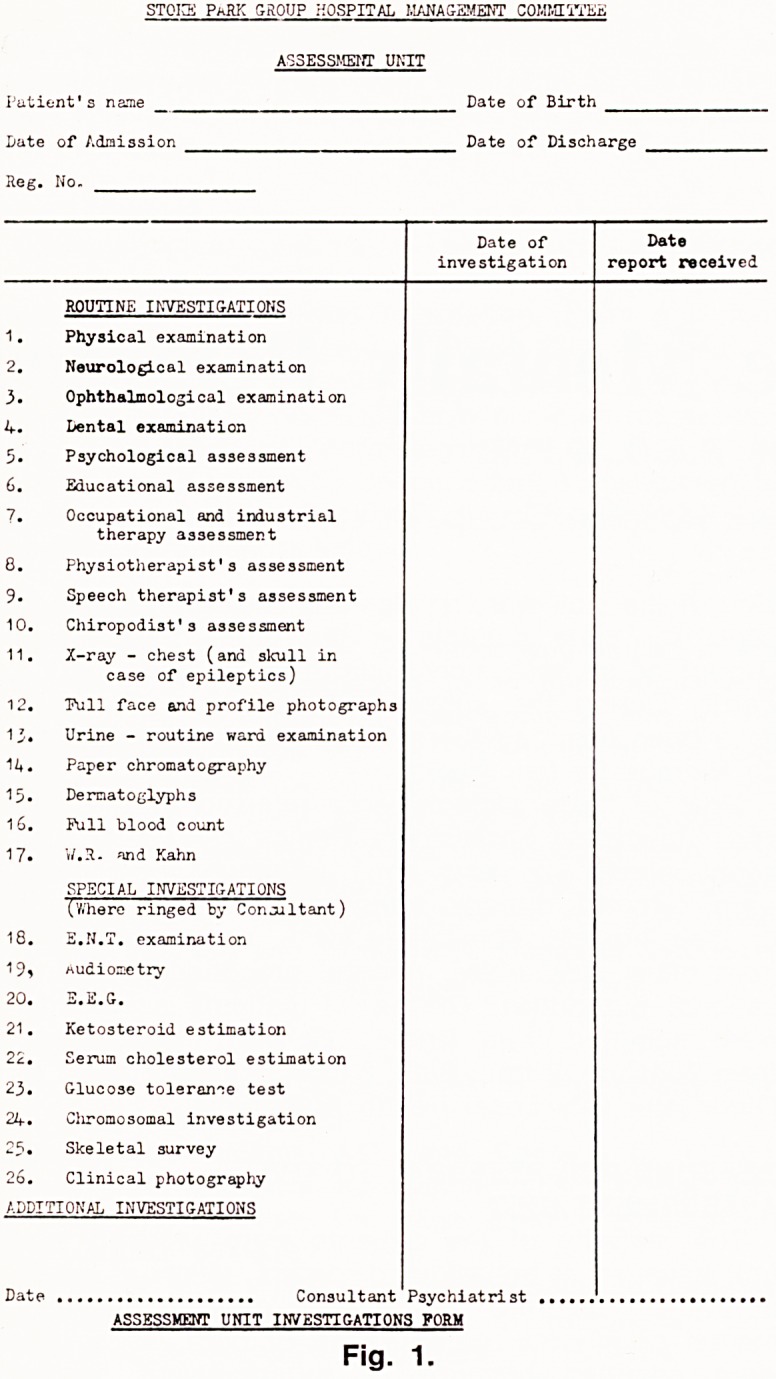


**Fig.2 f2:**
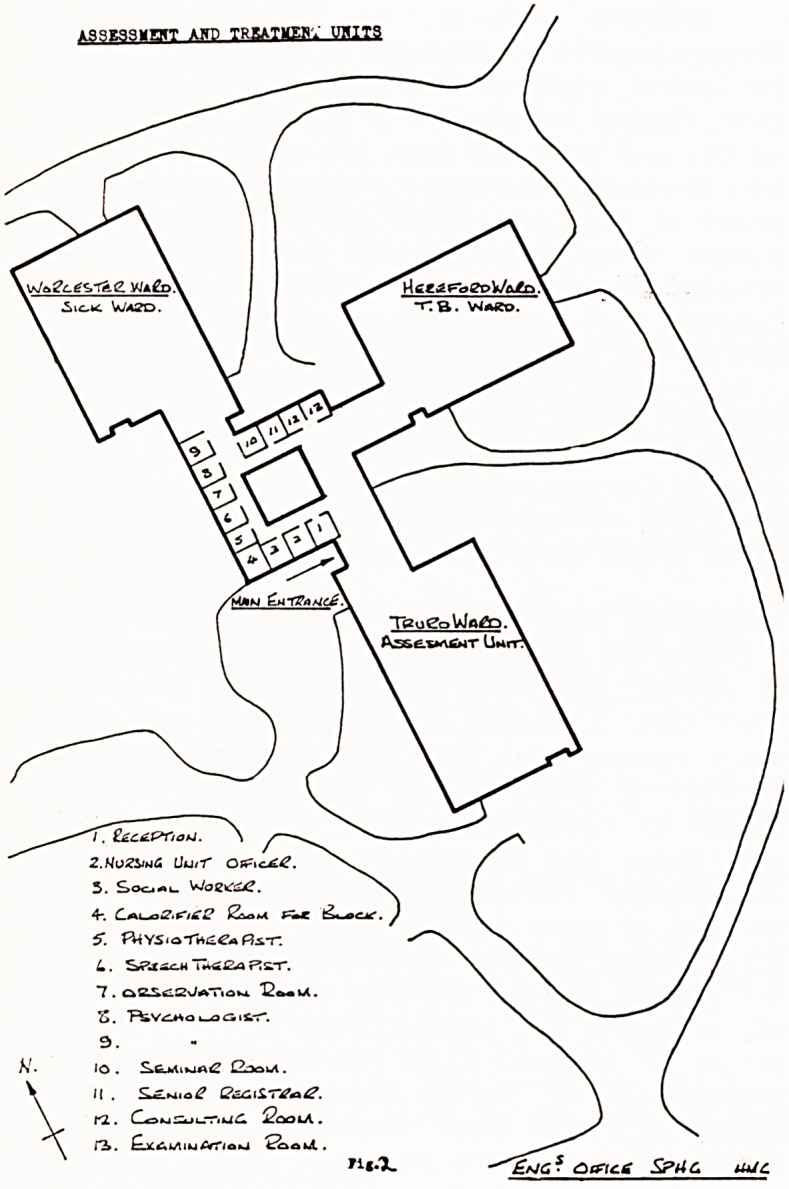


**Fig.3 f3:**